# Co-morbidity and drug treatment in Alzheimer's disease. A cross sectional study of participants in the Dementia Study in Northern Norway

**DOI:** 10.1186/1471-2318-11-58

**Published:** 2011-10-04

**Authors:** Fred Andersen, Matti Viitanen, Dag S Halvorsen, Bjørn Straume, Torgeir A Engstad

**Affiliations:** 1 Department of Community Medicine, University of Tromsø, (Breivika), Tromsø, (9037), Norway, and Árran Lulesami Centre, (Sentrum), Drag, (8270), Norway; 2 Department of Geriatrics, Karolinska Institutet, (Huddinge), Stockholm, (141 86) Sweden, and University of Turku, (Sirkkalankatu), Turku, (20520), Finland; 3 Department of Medicine, University hospital, (Breivika), Tromsø, (9038), Norway; 4 Department of Community Medicine, University of Tromsø, (Breivika), Tromsø, (9037), Norway; 5 Department of Geriatrics, University Hospital, (Breivika), Tromsø, (9038), Norway

## Abstract

**Background:**

Inappropriate medical treatment of co-morbidities in Alzheimer's disease (AD) is an increasing concern in geriatric medicine. The objective of this study was to compare current drug use related to co-morbidity between individuals with a recent diagnosis of AD and a cognitively healthy control group in a population based clinical trial in Northern Norway.

**Methods:**

Setting: Nine rural municipalities with 70 000 inhabitants in Northern Norway.

Participants: Participants with and without AD recruited in general practice and by population based screening.

187 participants with a recent diagnosis of AD were recruited among community dwellers. Of 791 respondents without cognitive symptoms, 500 were randomly selected and invited to further clinical and cognitive testing. The final control group consisted of 200 cognitively healthy individuals from the same municipalities. Demographic characteristics, data on medical history and current medication were included, and a physical and cognitive examination was performed. The statistical analyses were carried out by independent sample t-test, chi-square, ANCOVA and logistic regression.

**Results:**

A co-morbidity score was significantly higher in AD participants compared to controls. The mean number of drugs was higher for AD participants compared to controls (5.1 ± 3.6 and 2.9 ± 2.4 respectively, p < 0.001 age and gender adjusted), also when adjusted for co-morbidity. AD participants used significantly more anticholinergic, sedative and antidepressant drugs. For nursing home residents with AD the mean number of drugs was significantly higher compared to AD participants living at home (6.9 ± 3.9 and 4.5 ± 3.3, respectively, p < 0.001).

**Conclusions:**

AD participants were treated with a significantly higher number of drugs as compared to cognitively healthy controls, even after adjustment for co-morbidity. An inappropriate use of anticholinergic and sedative drugs was identified, especially among nursing home residents with AD. The drug burden and the increased risk of adverse reactions among individuals suffering from AD need more attention from prescribing doctors.

## Background

The proportion of elderly with age-related diseases is rapidly increasing worldwide representing a vulnerable population with respect to medication issues. In particular, this is true for Alzheimer's disease (AD), constituting 65 - 70% of all dementia subtypes, inflicting an extensive and serious impact on activities of daily living and quality of life for patients and their caregivers [1-5]. The prevalence of AD increases by age [6-8] as does a number of other age related disorders.

Individuals suffering from AD often have cardiovascular diseases such as coronary heart disease, stroke, diabetes mellitus and hypertension, requiring use of multiple drugs. Schubert et al. reported that patients with dementia attending primary care have on average 2.4 chronic conditions and receive 5.1 medications [9]. Likewise, psychiatric disorders like depression and sleeping disturbances are prevalent. Twenty-five to 35% of AD individuals have sleep disturbances being treated with hypnotics [10]. Particularly, anxiolytic-hypnotic agents, antidepressants and antihistamines that often exhibit central nervous system effects are associated with increased cognitive impairment, sedation and confusion. Consequently, multiple drug prescriptions are leaving elderly vulnerable to adverse reactions [10-12] and harmful interactions between psychotropic drugs and between psychotropic drugs and drugs aimed to treat co-morbidities, often classified as inappropriate drug prescriptions [13].

Despite the heavy drug load elderly are exposed to, few studies have examined overall medication in dementia [12], and even fewer studies have focused on appropriate medical treatment of co-morbidity in AD patients [14,15].

The main purpose of this paper is to compare drug treatment in relation to co-morbidity, focusing on inappropriate prescriptions between individuals with a recent diagnose of AD and a randomly selected cognitively healthy control group.

## Methods

### Participants

**Figure 1:**
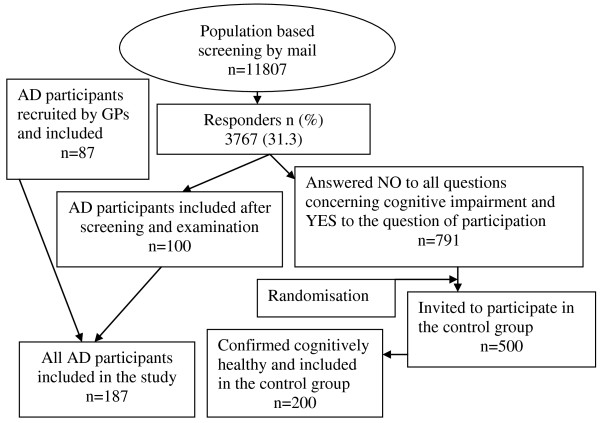
**Flowchart revealing recruitment methods and participants**.

From January 2006 to March 2008 187 participants with a recent diagnosis of a probable AD were included in The Dementia Study in Northern Norway, run in nine rural municipalities with 70000 inhabitants (11807 individuals > = 65 year). Forty-five AD participants were nursing home residents. The two different recruitment methods which were used and the baseline characteristics comparing the two samples are described in an earlier paper [16]. AD participants were recruited by general practitioners (n = 87) and by a population based screening (n = 100). The latter method also recruited a cognitively healthy control group (n = 200) (Figure 1). The present study is a cross sectional comparison between AD participants in a randomised controlled trial and a cognitively healthy control group.

### Clinical examination

All AD participants and the cognitively healthy controls passed the same examinations performed by trained physicians and nurses. Dementia and AD were diagnosed by GPs and geriatric specialists using the ICD-10 [17] criteria and the Statistical Manual of Mental Disorders fourth edition (DSM-IV-TR) [18]. Diagnostic discrepancies were discussed with a geriatric colleague and solved by consensus advised by National Institute of Neurological Disorders and Stroke-Alzheimer Disease and Related Disorders (NINCDS-ADRDA) [19] criteria for probable AD. A third specialist (MV) was consulted if disagreement continued. Blood pressure was measured automatically by DINAMAP ProCare [20]. Three consecutive blood pressures were recorded and the mean of the second and the third measures was used to calculate mean arterial blood pressure (MAP). Body mass index (BMI) was calculated. A 12 channels electrocardiogram (ECG) was registered. Cognition was tested at entry by Mini-Mental-State-Examination (MMSE) [21] and Clock drawing test [22]. Prior to the study onset two test technicians were trained at the Geriatric Department, University Hospital in Northern Norway. To improve intra- and inter-rater reliability they observed and evaluated each other by testing a number of patients with MMSE, Alzheimer's disease Assessment Scale, cognitive (ADAS-Cog) [23] and Neuropsychiatric Inventory (NPI) [24].

### Medical history

The demographic characteristics and self-reported medical history were registered in a questionnaire regarding the most common life style and age related illnesses like cardiovascular diseases (i.e. myocardial infarct, angina pectoris, congestive heart failure, atrial fibrillation), hypertension, stroke, diabetes, chronic pain and neuropsychiatric complains. A co-morbidity score was calculated for each participant by adding the number of age related diseases identified as AD risk factors [25,26] providing a sum score of chronic health conditions [9]. The physicians had access to the medical record of AD participants confirming given information. In addition the caregiver or a next of kin was encouraged to extend the medical history. Computerised ECG assessed by an experienced physician validated a medical history of coronary heart disease.

### Drug treatment

Drugs used at study entry by AD participants and controls were registered and daily medication was checked according to medication charts, information given by caregiver(s) and the medical record. The medication charts were also checked against reported co-morbidities. Drugs were classified and recorded according to the Anatomic Therapeutic Classification system (ATC codes) [27] like anxiolytic-hypnotics (N05B and C) antidepressants (N06A), antipsychotics (N05A), lipid lowering agents (C10A), antihypertensive drugs (C09A, B, C and D, C08C and D, C07A and B, C03A, C, D and E) and a heterogeneous group consisting of drugs with anticholinergic activities. According to the Anticholinergic Drug Scale [28] drugs with anticholinergic activity are grouped into four levels (level 0 - 3) [28-30]. In this study drugs exhibiting a significant or a moderate level of anticholinergic effect (level 2 and 3) were recorded. Inappropriate drugs were classified according to the Screening Tool of Older Persons' Prescription (STOPP) criteria which comprise a list of drugs at risk of interaction and adverse reactions when combined with common illnesses in geriatric practice [13].

### Approvals

The Dementia study in Northern Norway complies with the Norwegian Research Legislation and the Helsinki Declaration, and the present manuscript complies with the CONSORT statement. The Regional Committee for Medical Research Ethics in Northern Norway, The Privacy Ombudsman for Research, The Directory of Health and Social Welfare and The Norwegian Medicine Agency including registration in the EudraCT database (no 2004-002613-37) approved the study. Each AD participant gave a written informed consent co-signed by a next of kin or a caregiver whereas participants in the cognitively healthy control group gave a written informed consent on their own. The present manuscript is based on data already collected in The Dementia Study in Northern Norway.

### Statistics

Statistical analyses were carried out using SPSS version 18.0 (SPSS Inc. Chicago, US). Differences in age, gender, heredity, co-morbidity and current drug use between participants with and without AD were analyzed by independent sample t-tests and Chi-Square tests. Adjustment for age, gender and co-morbidity score were done by covariance analyses and logistic regression. A two-sided 5% significance level was used. The results are presented both unadjusted and age and gender adjusted. The calculation of 95% confidence interval (CI) refers to age and gender adjusted differences between samples or groups according to the ANCOVA or to logistic regression analyses.

## Result

### Baseline characteristics

AD participants were older (p < 0.001), more often female (p < .0.001) and reported dementia among close relatives (p < 0.001) more frequently compared to controls. Mean arterial blood pressure was significantly lower among AD participants compared to controls (p = 0.009 age and gender adjusted) (Table 1).

**Table 1:** Baseline characteristics of AD participants compared to controls

	AD n = 187	Control n = 200	p-value
Age (years ± SD)			
	80.9 ± 7.0	72.5 ± 5.5	< 0.001
Gender, women			
n (%)	113 (60)	85 (43)	< 0.001
Familiar disposition			
n (%)	56 (31)	29 (15)	< 0.001
Education ≥ 10 years			
n (%)	33 (17)	126 (63)	0.33*
MMSE score ± SD			
	23.1 ± 4.5	28.7 ± 1.6	< 0.001*
MAP ± SD			
	100.9 ± 16.6	107.2 ± 13.6	0.009*
BMI ± SD			
	25.4 ± 5.0	26.0 ± 4.2	0.59*

*Age and gender adjusted. MAP = Mean arterial blood pressure. BMI = Body mass index. SD = standard deviation

### Medical history

The co-morbidity score was higher in AD participants compared to controls (2.1 ± 1.5 and 1.3 ± 1.2 respectively, p < 0.001). AD participants had a higher frequency of cardiovascular diseases (i.e. angina pectoris, myocardial infarct, congestive heart failure and atrial fibrillation), stroke, diabetes mellitus, hypertension, chronic obstructive bronchitis and chronic pain but significantly for chronic obstructive bronchitis only (adjusted for age and gender) (Table 2). The differences in mean number of co-morbidities were non-significant between AD participants recruited by screening or by GPs (2.3 ± 1.5 and 2.0 ± 1.6 respectively, p = 0.20) and between AD participants living at home or in nursing homes (2.4 ± 1.6 and 2.1 ± 1.5 respectively, p = 0.20). (Data not shown)

**Table 2:** Co-morbidities AD participants compared to controls

	AD participants n = 187	Controls n = 200	Unadjusted p-value	Adjusted p-value*
Co-morbidity score				
	2.1 ± 1.5	1.3 ± 1.2	< 0.001	< 0.001
Cardiovascular diseases**				
n (%)	139 (74)	114 (57)	< 0.001	0.14
Angina pectoris				
n (%)	48 (26)	18 (9)	< 0.001	0.37
Myocardial infarct				
n (%)	27 (14)	24 (12)	0.52	0.26
Atrial fibrillation				
n (%)	34 (18)	18 (9)	0.008	0.40
Stroke				
n (%)	33 (18)	11 (6)	< 0.001	0.82
Hypertension				
n (%)	102 (55)	82 (41)	0.008	0.14
Diabetes mellitus				
n (%)	21 (11)	17 (9)	0.37	0.15
Chronic obstructive bronchitis				
n (%)	19 (10)	10 (5)	0.054	0.003
Chronic pain				
n (%)	47 (25)	41 (21)	0.28	0.17

* Adjusted for age and gender. ** Participants suffering from one or more of congestive heart failure, angina pectoris, myocardial infarct, angina pectoris or atrial fibrillation.

### Drug treatment

The mean number of drugs was significantly higher in AD participants compared to controls (5.1 ± 3.6 and 2.9 ± 2.4 respectively, age and gender adjusted difference of means 1.48, 95%CI 0.78 to 2.21, p < 0.001). This finding remained unchanged when adjusting for co-morbidity score (p < 0.001). Forty-eight percent of AD participants used five or more drugs compared to 23% in the control group (95%CI for age and gender adjusted differences 0.076 to 0.298, p = 0.001) (Table 3). AD participants used a greater number of antihypertensive drugs. Inappropriate drugs such as anticholinergics, antidepressants and anxiolytic/hypnotics were prescribed more frequently to AD participants compared to controls (Table 3).

**Table 3:** Current drug use in AD participants compared to controls

	AD n = 187	Control n = 200	Difference*	95%CI*	p-value*
Mean number of drugs ± SD					
	5.1 ± 3.6	2.9 ± 2.4	1.48	0.78 to 2.21	< 0.001
Mean number of antihypertensive drugs ± SD					
	1.4 ± 1.3	0.8 ± 0.9	0.28	0.012 to 0.538	0.040
Participants using five drugs or more					
n(%)	90(48)	46(23)	0.19	0.076 to 0.289	0.001
antihypertensive drugs					
n(%)	128(68)	101(50)	0.039	-0.074 to 0.153	0.50
anticholinergic drugs					
n(%)	43(23)	12(6)	0.161	0.080 to 0.243	< 0.001
antidepressants					
n(%)	24(13)	4(2)	0.091	0.030 to 0.151	0.004
anxiolytic-hypnotic drugs					
n(%)	42 (22)	15(8)	0.090	0.008 to 0.172	0.032
inappropriate drugs**					
n(%)	69(37)	22(11)	0.193	0.097 to 0.288	< 0.001
lipid lowering agents					
n(%)	53(28)	69(35)	0.000	-0.111 to 0.112	0.99

SD = Standard deviation. CI = Confidence interval for age and gender adjusted differences. * Age and gender adjusted. Percentage differences in CIs are expressed in fractions. **According to STOPP criteria for inappropriate drug treatment

The total number of prescribed drugs was significantly higher among nursing home residents with AD (n = 45) compared to AD participants living at home (6.9 ± 3.9 and 4.5 ± 3.3 respectively, age and gender adjusted differences of means -2.07, 95%CI -3.30 to -0.83, p = 0.001). Nursing home residents with AD used significantly more antidepressants (13 of 45 and 11 of 142 respectively, p < 0.001) and anxiolytic-hypnotics (18 of 45 and 23 of 141, p < 0.001) compared to AD participants living at home. No significant differences in number of drugs was detected between AD participants when MMSE score was grouped as a dichotomized variable (= < 21 or > 21) (5.8 ± 4.2 versus 4.8 ± 3.2 respectively, p = 0.16, age and gender adjusted) or between AD participants recruited by screening or by GPs (5.0 ± 3.5 versus 5.2 ± 3.7 respectively, p = 0.56 age and gender adjusted) (Data not shown).

## Discussion

### Medical history

The co-morbidity score was significantly higher in AD participants compared to controls but turned out non-significant for nine of ten recorded co-morbidities when adjusting for age and gender. This is in line with other studies[9,31,32] whereas one study using a historical cohort of community dwellers with and without AD reported a significant higher prevalence for 12 of 14 health conditions among AD individuals [33].

In our study, AD participants reported higher lifetime occurrence of hypertension and were more often treated with a greater number of antihypertensive drugs. Previous observations have shown that AD is associated with elevated systolic blood pressure in midlife followed by a greater decrease with aging compared to cognitively healthy individuals [31,34]. Midlife hypertension may generate arteriosclerosis, cerebral small-vessel disease and disturbed cerebral autoregulation in resistant arteries [35-37], leaving elderly AD individuals prone to cerebral hypoperfusion and cognitive worsening due to extensive antihypertensive treatment [38,39].

### Drug treatment

In the present study AD participants consumed a significant higher number of drugs compared to controls, similar to the results reported by Schubert et al [9]. Adjusting for co-morbidity score did not change the results. Any prescription has a potential risk of adverse reactions and the risk increases with the number of drugs, from a 10% with one drug to 75% with five or more drugs [40]. In our study 48% of the AD participants used five or more drugs compared to 23% of the controls. The nursing home AD residents consumed a mean of 6.9 ± 3.9 different drugs a day, quite similarly to what Holmes et al reported [41].

Inappropriate medication is based on the STOPP criteria [13], and in our study 37% of the AD participants used one or more drugs considered inappropriate compared to 11% in the control group (p < 0.001) (Table 3). In a recent paper Barnett et al concluded that the high prevalence of inappropriate drugs in older people continues to occur despite the recognition and concerns of iatrogenic harms [42].

In the present study AD participants were significantly more often treated with drugs with anticholinergic side effects compared to controls (p < 0.001). Drugs with anticholinergic properties are considered inappropriate in elderly patients as a consequence of adverse reactions including constipation, dry mouth, blurred vision and dizziness which may contribute to falls and delirium [43]. Increased levels of anticholinergic activity are associated with increased cognitive decline assessed by MMSE and even dementia [11,44-46]. Interruption of the anticholinergic medication may represent a therapeutic option to improve cognitive performance [47], especially where anticholinergic drugs are give simultaneously with cholinesterase inhibitors.

In our study, anxiolytic-hypnotics and antidepressants were used more frequently in the AD group compared to the control group (p = 0.032). Among nursing home AD residents 18 of 45 (40%) used anxiolytic-hypnotics. In another study 20% of AD individuals in general practice were prescribed at least one psychotropic drug. Anxiolytic-hypnotic drugs are known to influence alertness, power of reaction, risk of falls and functional and cognitive impairment [48].

### Strengths and weaknesses

This study has a population-based design and is accomplished on a community level providing a homogenous sample with minimal environmental influence. The participants in this study were examined according to standardized procedures and diagnoses were based on accepted validated criteria and were confirmed by an expert panel. A self-report of the medical history by AD participants could be inaccurate and at risk of recall bias, but the method is in accordance with several geriatric studies based on self-reported chronic medical conditions [40].

A weakness of the study is that defined daily doses of medication and the length of treatment have not been recorded. The differences in mean age and gender distribution between the AD group and the control group are significant, but adjustment for age and gender was performed in the statistical analyses. However, age and gender adjustment is questionable when it comes to some of the variables like familiar disposition and prevalence of hypertension.

## Conclusion

In the present study AD participants used more drugs than cognitively healthy controls despite similar frequency of co-morbidity. The AD participants had nearly a two-fold use of drugs and inappropriate use of anticholinergic, anxiolytic-hypnotic and antidepressants were detected. The drug burden and the increased risk of adverse reactions among individuals suffering from AD need more attention from prescribing doctors.

## List of abbreviations

AD: Alzheimer's disease; ADAS-Cog: Alzheimer's disease Assessment Scale, cognitive (Scale 0-70, increasing disability with increasing score); ATC: Anatomic Therapeutic Classification system; BMI: Body Mass Index; CI: Confidence interval; DSM-IV-TR: Statistical Manual of Mental Disorders fourth edition; ECG: Electrocardiogram; GP: General Practitioner; ICD-10: International classification of diseases 10^th ^Revision; MAP: Mean arterial blood pressure; MMSE: Mini-Mental State Examination (Scale 0-30, better function with increasing score); NPI: NeuroPsychiatric Inventory (Scale 0-144, increasing disability by increasing number); NINCDS-ADRDA: National Institute of Neurological Disorders and Stroke-Alzheimer Disease's and Related Disorders; SD: Standard deviation; STOPP: Screening Tool for Older Persons' Prescription.

## Competing interests

The authors declare that they have no competing interests.

## Authors' contributions

FA has initiated, coordinated and conducted this study in close co-operation with the scientific advisory board at The University of Tromsø. He has examined and diagnosed patients recruited both in general practice and by postal cognitive screening. He is also responsible for analyzing baseline data, the main results of the study and the cross sectional analyses referred in the present paper. MV is a member of the scientific advisory board and has participated in planning of the present study and in revising this manuscript. DSH has contributed significantly in drafting and writing of this paper. BS participated in the planning of the study, supervising implementation and analysis and has revised the manuscript. He is a member of the scientific advisory board. TE has been the main supervisor and member of the scientific advisory board, participating in all stages of this study; - planning, lecturing, collecting data, discussing results and writing. All authors have full access to all the data (including statistical reports and tables) and have approved the final version of the paper.

## Pre-publication history

The pre-publication history for this paper can be accessed here:

http://www.biomedcentral.com/1471-2318/11/58/prepub
